# Obstetric fistula in low-resource countries: an under-valued and under-studied problem – systematic review of its incidence, prevalence, and association with stillbirth

**DOI:** 10.1186/s12884-015-0592-2

**Published:** 2015-08-26

**Authors:** Karen D. Cowgill, Jennifer Bishop, Amanda K. Norgaard, Craig E. Rubens, Michael G. Gravett

**Affiliations:** College of Nursing, Seattle University, Seattle, WA USA; Department of Global Health, University of Washington, Seattle, WA USA; Global Alliance to Prevent Prematurity and Stillbirth, Seattle Children’s, Seattle, WA USA; Department of Obstetrics and Gynecology, University of Washington, Seattle, WA USA

**Keywords:** Vaginal fistula, Obstetric labor complications, Stillbirth, Developing countries

## Abstract

**Background:**

Obstetric fistula (OF) is a serious consequence of prolonged, obstructed labor in settings where emergency obstetric care is limited, but there are few reliable, population-based estimates of the rate of OF. Stillbirth (SB) is another serious consequence of prolonged, obstructed labor, yet the frequency of SB in women with OF is poorly described. Here, we review these data.

**Methods:**

We searched electronic databases and grey literature for articles on OF in low-resource countries published between January 1, 1995, and November 16, 2014, and selected for inclusion 19 articles with original population-based OF incidence or prevalence data and 44 with reports of frequency of SB associated with OF.

**Results:**

OF estimates came from medium- and low-HDI countries in South Asia and Africa, and varied considerably; incidence estimates ranged from 0 to 4.09 OF cases per 1000 deliveries, while prevalence estimates were judged more prone to bias and ranged from 0 to 81.0 OF cases per 1000 women. Reported frequency of SB associated with OF ranged from 32.3 % to 100 %, with estimates from the largest studies around 92 %. Study methods and quality were inconsistent.

**Conclusions:**

Reliable data on OF and associated SB in low-resource countries are lacking, underscoring the relative invisibility of these issues. Sound numbers are needed to guide policy and funding responses to these neglected conditions of poverty.

**Electronic supplementary material:**

The online version of this article (doi:10.1186/s12884-015-0592-2) contains supplementary material, which is available to authorized users.

## Background

Prolonged obstructed labor is common where emergency obstetric care is unavailable or inaccessible, and can lead to a host of physical and psychosocial injuries, collectively known as “obstructed labor injury complex” [1]. For women who survive prolonged, obstructed labor, obstetric fistula (OF) is the most severe of these. OF is a life-altering birth injury caused when the presenting fetal part continually compresses the birth canal tissues, bladder base, urethra, or sometimes rectum, causing ischemia and necrosis of the tissues, resulting in a fistula. In most cases, the fistula occurs between the vagina and bladder (i.e., vesicovaginal), but it may also occur between the vagina and rectum (i.e., rectovaginal) [2]. As a result of the fistula, women leak urine and/or feces out of the vagina continually without control, and can experience medical complications, including infection [3]. Underdeveloped pelvic bony structure is a risk factor for obstructed labor and obstetric fistula. In regions where young girls become pregnant, or where malnutrition that leads to stunting is prevalent, obstructed labor and obstetric fistula are more common [1, 4]. Women with OF also suffer significant psychosocial repercussions, including isolation, divorce, loss of social roles -- including the role of mother, for those whose infants are stillborn, loss of income, stigmatization, shame and diminished self-esteem [5].

In countries where emergency obstetric care is available and accessible, OF has been virtually eliminated. However, it continues to be prevalent and problematic in many less-developed regions of the world despite the fact that it is preventable and treatable [6]. Surgical repair has a success rate of almost 90 %, but can be difficult for women to access or afford [7].

Many women who experience OF are also left to grieve a stillborn baby [5]. SB rates are underreported and under-valued [8, 9], but are known to be closely associated with maternal mortality rates [10]. Little has been reported about the association of SB with maternal morbidity rates, which may be 10 to 100 s of times higher than maternal mortality rates [2, 11]. Between 2.14 and 3.82 million SB, ¾ of which were in south Asia and sub-Saharan Africa, are estimated to have occurred in 2009 [12]. When labor is prolonged for days without appropriate emergency obstetric care, fetal death may result; as many as one third of SB occur in the intrapartum period [10], indicating that the fetus might have survived if adequate obstetric care had been received. Fetal death during labor is as many as 50 times higher in developing than developed countries [13]. The treatment most likely to improve both maternal and fetal outcomes in obstructed labor is cesarean section or instrumental delivery, so increasing access to emergency obstetric care is a cornerstone of preventing both OF and SB [14, 15].

While some groups have worked to find, treat, and prevent OF in low-resource countries, system-wide responses are lacking, as are reliable population-based estimates of the incidence and prevalence of OF [2, 16, 17] and of the correlation between stillbirth and OF. These data are needed to inform policy and to make visible the costs in human suffering and potential lives lost from lack of effective emergency obstetric care. This paper reviews the literature reporting original population-based estimates of OF incidence and prevalence rates in low-resource countries and assesses their precision and risk of bias; in the absence of population-based data linking OF and SB, it also reports facility-based estimates of SB in births that caused OF.

## Methods

### Search strategy

**Fig. 1:**
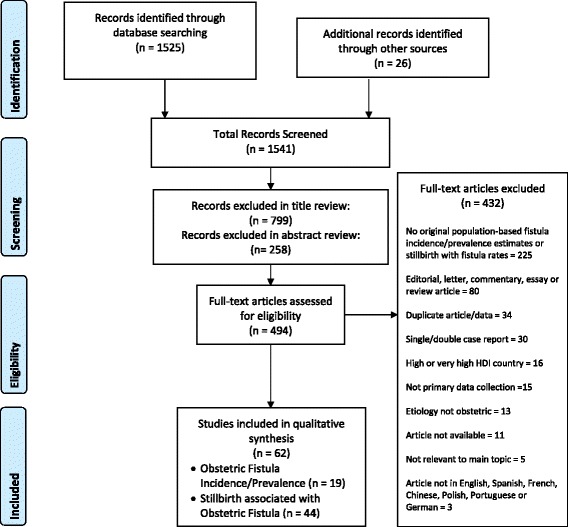
PRISMA Flow diagram: Selection of studies for inclusion in review

See Additional file 1 for the study’s PRISMA checklist. We conducted electronic searches for articles on OF using PubMed/MEDLINE and the CAB Global Health Database; results were restricted to articles published between January 1, 1995 and November 16, 2014. Complete search terms are available as Additional file 2; no protocol for the review was registered (see PRISMA item 5). We reviewed titles and/or abstracts of all search results, and selected articles addressing obstetric fistula incidence, prevalence, or the correlation between obstetric fistula and stillbirth in low-resource countries for full-text review. An ancestry search of references in reviewed articles and email requests for grey literature to researchers, health administrators, and clinicians in target countries yielded additional resources (see Fig. 1).

### Inclusion and exclusion criteria

Published or grey literature articles obtained through ancestry searches or in response to email requests were included if they were published on or after January 1, 1995 and met the following inclusion criteria: 1) they provided original population-based OF incidence and/or prevalence data or frequency of SB associated with OF, 2) data were from high-, medium-, or low- (but not very high-) Human Development Index (HDI) countries and territories as defined by the United Nations Development Programme [18], and 3) the resource was in English, Spanish, French, Chinese, Portuguese, Polish, or German.

### Data extraction

Reviewers (KDC, AKN, JB) independently extracted data on OF, including country or region, study period, study design, description of study participants, data source, number of fistula cases, fistula incidence/prevalence estimates, and on SB, including number of stillbirth cases and percentage of stillbirth with fistula. We standardized fistula incidence or prevalence estimates to be expressed per 1000 to facilitate comparisons, and obtained exact 95 % confidence intervals using OpenEpi.com, a free open-access tool that permits simple epidemiologic calculations.

### Assessment of bias

We assessed risk of bias in individual OF studies by taking into account the study design, clarity of the documentation of methods, the definition of the reference population and the precision of the estimate of its magnitude, whether samples were selected randomly, the case definition of OF applied, whether cases were determined based on self-report alone or on physical exam, and peer-review status. For studies reporting the proportion of SB among births that led to OF, we did not perform an explicit assessment of bias, as these estimates were generally not part of the studies’ main objectives. Instead, we commented on factors that might decrease the validity of estimates, recognizing that small sample size, while it may decrease precision of estimates, is not in itself a source of bias.

## Results

The literature search produced 62 articles that met the inclusion criteria (see Fig. 1). Nineteen provided population-based obstetric fistula incidence and/or prevalence data (7 from South Asia, 12 from Africa, including 2 reporting on the same study [19, 20]) and 44 provided stillbirth rates associated with the birth that caused an obstetric fistula; one article [21] provided both stillbirth data and population-based obstetric fistula incidence/prevalence data.

### Obstetric fistula incidence/prevalence

Additional file 3: Table S1 describes the included obstetric fistula incidence/prevalence studies. Obstetric fistula incidence and/or prevalence data were available for three countries in South Asia, two classified as medium HDI and one as low HDI, and for ten countries in Africa and for the West African region, all classified as low HDI. Of the five population-based incidence estimates, four were considered to have low risk of bias, and one to have a moderate-high risk of bias. This latter study yielded the highest incidence estimate, which was derived from a model based on Demographic and Health Survey (DHS) data from Nigeria and projected 4.09 OF cases per 1000 deliveries in women under 20 years, and 2.11 per 1000 deliveries in women aged 12–49 years [4]. This model extrapolated OF incidence using data about the frequency of prolonged labor and applying a probability of obstruction given prolonged labor and a probability of fistula given obstruction. Among the other studies estimating OF incidence, a research group in West Africa reported 0.1 OF cases per 1000 deliveries based on physical exams of post-partum women [19, 20], with a higher incidence in rural than urban residents (1.2 (95 % confidence interval (CI) 0.15–4.46) vs. 0 (95 % CI 0–0.18) cases per 1000 among post-partum women). Two studies from south Asia [22, 23] first asked 1-12-month post-partum women whether they had constant leaking of, in one case, feces, and in the other, either feces or urine, from the vagina; this yielded symptom incidence rates of 5.19 (95 % CI 0.87–17.05) and 5.39 (95 % CI 1.37–14.59) per 1000. However, a physical exam to confirm that the symptoms were due to OF yielded only 2.60 (95 % CI 0.13–12.74) and 0 (95 % CI 0–5.37) OF cases per 1000 post-partum women. A third report from south Asia found 0 (95 % CI 0–2.6) OF cases per 1000 post-partum women evaluated by physical exam [24].

Period or lifetime prevalence estimates were judged more prone to bias than incidence estimates, with 9 of the 13 prevalence estimates classified as having a moderate, moderate-high, or high risk of bias. Prevalence estimates varied widely, with those obtained by asking women whether they had experienced leakage of urine or feces from the vagina following a delivery consistently higher (10.60-81.0 cases per 1000) [25–29] than those based on physical exam or hospital records (0–4.5 OF cases per 1000) [21, 30–34]. The reference populations for estimates differed, with most based on women of reproductive age, defined as 15 to 44, 49, or 54 years, and some limited to women who had ever married or ever been pregnant. One grey literature report [35] did not give detailed information about the sample or the questions by which OF was assessed. A recent, high-quality population-based cross-sectional study reported 4.5 (95 % CI 2.8–6.8) OF cases per 1000 parous women aged 15 or older in rural Pakistan [33].

### Stillbirth with obstetric fistula

Additional file 4: Table S2 describes data from the 44 studies that reported SB data. The reported proportion of infants stillborn to women who developed OF at that birth ranged from 32.3 % to 100 %. In the 3 studies with sample sizes greater than 500, the proportions were 87.5 % (n = 1,243) [36], 91.7 % (n = 899) [37], and 92.2 % (n = 14,822) [34]. The majority of the studies were done solely in Africa, while two studies reported on India [38, 39], and one included information about both Africa and Bangladesh [36].

Three articles did not report if SB was the outcome of the OF-inducing delivery or a previous one [40–42]. Two articles reported a large percentage of unknown fetal outcomes; one of these [43] reported a SB proportion of 55 %, with an additional 38 % of unknown outcomes, while the other [44] reported 46 % stillborn with fistula and 45 % with unknown fetal outcome; it is likely that some deliveries with unknown outcomes also resulted in stillbirths. Another article [45] did not report fetal outcome, but noted that in 21 women (56 % of cases), “the mode of delivery associated with fistula was ‘destructive delivery’ (evacuation of a stillborn fetus).” Fetal outcome of the remaining 44 % of cases was not reported.

In one study that reported SB statistics for both cesarean section deliveries and spontaneous vaginal deliveries [46], SB rates were higher in the vaginal delivery groups (96.4 %) than the cesarean group (87.2 %). In another study of nearly 15,000 women with OF, more than half of infants were male (635 cases, 70.6 %), and SB rates were higher for boy (91.9 %) than girl (78.9 %) fetuses [34].

## Discussion

Reported rates of OF vary widely; some of the variations represent true differences in incidence, while others are artifacts of study design. Studies aiming to provide population-based data on OF are available from only a small number of countries in Africa and South Asia, underscoring the relative invisibility of this problem to policymakers and funders.

OF occurs more often in rural areas [20] and may be hidden from view, as those afflicted often experience shame and isolation from their communities [47]. Many women who might have developed OF had they survived a difficult childbirth instead die, so are not included in population-based estimates [48]. Reaching rural women is a daunting task, and they are at higher risk for labor complications [28]; however, most research is facility-based, accounting only for women who are able to access health care.

Where health systems are weak and vital registration systems spotty or nonexistent, policymakers and health service providers do not have surveillance data to track vital events and measure population size [49]. Reliable population-based estimates of OF and associated SB are needed to guide and evaluate prevention and treatment programs, but population-based studies are difficult to conduct. Studies of OF prevalence are more prone to bias than studies of incidence; studies of incidence may be based on cohorts of pregnant or post-partum women that can be reliably followed over a defined period, while studies of prevalence require surveying or examining all parous women in a population. Both are complicated by the relative rarity of OF [17]. The few population-based OF incidence and prevalence estimates that are available have used different definitions of fistula as well as different methods of sampling and case ascertainment. For example, several of the estimates we report here are based on DHS interviews, which, as a proxy measure of OF, asked parous women whether they had experienced uncontrollable leakage of urine or stool from the vagina [28]. The DHS estimates are much higher than estimates based on other definitions of OF, and in fact, in studies where women were both asked if they had symptoms of OF and also examined, the frequency of OF on exam was lower than by self-report [22, 23, 33]. Thus, physical examination is required to reliably establish OF. Tunçalp, et al. [50] reported a positive predictive value of the 2008 Nigeria DHS questions of only 47 % in a subsample of women who presented for fistula screening; the predictive value would be much lower in the general population, where the prevalence of OF would be lower. We concur with Stanton [16], Wall [2], and Zheng [51] that current published estimates of OF incidence and prevalence are unreliable and do not support the conduct of a meta-analysis given the poor quality of the data.

In the absence of reliable data, an interesting approach some authors have taken is to estimate OF incidence using estimated probabilities of OF given obstructed labor and of obstructed labor given prolonged labor, and then applying these to prolonged labor data [2, 4]. However, even the best estimates cannot replace hard data; in response to the paucity of reliable data, in 2007 Stanton, et al. proposed a series of questions to be added to the DHS that would be more specific for OF incidence and prevalence and that would also capture cases in deceased siblings of survey respondents [16], thus generating reliable, comparable population-based estimates and avoiding the survival bias resulting from collecting data only on living subjects. After our review was completed, Maheu-Giroux, et al. published a meta-analysis of DHS and Multiple Indicators Cluster Surveys (MICS) data (grey literature reports) on vaginal fistula (VF) collected between 2005 and 2013 from nineteen sub-Saharan African countries [52]. In this excellent paper based on rigorous analysis using a hierarchical Bayesian approach, the authors’ best estimates of lifetime and point VF prevalence per 1000 women aged 15–49 years in these countries was 3.0 cases (95 % credible interval (CrI) 1.3–5.5) and 1.0 case (95 % CrI 0.3–2.4), respectively. However, they caution that data from these surveys may include false positive cases, since the surveys are not followed by gold-standard gynecological exams. Following these surveys with physical exams where feasible, as Adler, et al. [32] did in a study using a similar approach, would provide a measure of their validity and improve data quality even further. House-to-house studies that systematically enumerated female residents aged 10 and above while actively searching for cases, then confirmed cases by physical exam (and, ideally, offered fistula repair surgery), could be a viable tool for reliably establishing population prevalence in an area. Adding information about birth outcomes could help to better quantify the association between OF and SB.

SB rates are high in women who develop OF, but estimates of the proportion of cases in which the two co-occur are variable and imprecise. Nonetheless, the association between OF and SB is clear, with most women with OF reporting a stillbirth. While we can’t say for sure how often OF and associated stillbirth occur, we do know, to a large extent, why they occur. The reasons are systemic: prenatal care is difficult to access because of cost, availability, and/or accessibility [53]; cultural expectations to give birth at home without assistance, or with an unskilled birth attendant, may be at odds with programs to promote facility-based births and/or skilled birth attendance [28, 37, 43]; emergency obstetrical facilities and care, when they exist, are often inadequate, and impediments to treatment, such as waiting for permission to seek care, lack of transportation, desire to try traditional treatments, unawareness of available services, or distance from health care facilities, may limit their use [43]. Fetal and neonatal deaths that occur at home are often not reported [13]. Stillbirth may be more common with male babies, perhaps because male fetuses are larger on average [37].

We did not attempt a meta-analysis because nowadays, OF is not an outcome that occurs at a consistent rate around the world or even within regions. OF is an indicator of weak emergency obstetric care systems [54], and its occurrence is variable at national and subnational levels. Furthermore, there were inconsistencies in definition of fistula, as noted above, and some fistulas may not have been obstetric in origin. Risk of bias was moderate or high in most studies, especially those in grey literature. Study populations were variably defined as women who had ever borne children, or recently borne children, or who were of childbearing age – excluding older women who might no longer be fertile but could still suffer fistula, as noted in a community-based screening in Nigeria [55]. One study inexplicably excluded women with repaired fistula from its estimate of lifetime OF prevalence [29]. There was incomplete reporting of birth outcomes in some studies reporting on SB. Many studies did not differentiate between SB and early neonatal mortality, which may have artificially increased or decreased the SB rates associated with the birth that caused the fistula, since these early neonatal deaths might also have been linked to the obstructed labor that caused the OF.

Aside from the risk of bias inherent in the studies themselves, an important limitation of this review is that the search strategy we used may not have been sensitive enough to capture studies of reproductive morbidity in which fistula was neither explicitly sought nor found, but where it would have been reported had it been found. As Adler, et al. [56] point out, excluding studies with negative findings when attempting to generate an overall estimate of OF rates constitutes search bias [57]. We did not attempt to generate an overall estimate of OF rates, but rather to assess the internal validity of estimates of OF where it was recorded, so we do not feel that the possible omission of studies with negative findings weakens our review. We did, nonetheless, examine the references cited by Adler, et al. and include four that met our inclusion criteria but were not returned by our search strategy. A strength of our review is that we calculated 95 % confidence intervals around all estimates to illustrate their inherent variability, and as a reminder that zero is not always zero: with small sample sizes, an event that occurs on the order of <5 times per 1000 births could easily be missed.

## Conclusions

In summary, OF remains a significant obstetrical problem in low-resource countries. It is strongly associated with stillbirth, as both are related to obstructed labor in the absence of emergency obstetrical care. Reliable data on OF and associated SB in low-resource countries are lacking, underscoring the relative invisibility of these issues; sound numbers are needed to guide policy and fund responses to these neglected conditions of poverty.

## Additional files

Additional file 1:
**PRISMA checklist.**
Additional file 2:
**Search strategy.**
Additional file 3: Table S1. Obstetric fistula incidence/prevalence estimates by country [58, 21]. Additional file 4: Table S2. Obstetric fistula with stillbirth data [59–86].

## Abbreviations

DHS: Demographic and health survey
HDI: Human development index
OF: Obstetric fistula
PRISMA: Preferred reporting items for systematic reviews and meta-analyses
SB: Stillbirth
