# Supplementary material for: Obstetric fistula in low-resource countries: an under-valued and under-studied problem – systematic review of its incidence, prevalence, and association with stillbirth
Source: BMC Pregnancy Childbirth. 2015 Aug 26;15:193. doi: 10.1186/s12884-015-0592-2 (PMC4550077; doi:10.1186/s12884-015-0592-2)
Supplement: Additional file 2: — Search strategy. [file 12884_2015_592_MOESM2_ESM.docx]

Text S3:

Search Terms

PubMed/MEDLINE search terms:

((vaginal OR vesicovaginal OR rectovaginal OR obstetric OR ureterovaginal OR urethrovaginal OR genitourinary OR urogenital) AND (fistula OR fistulas OR fistulae)) NOT (iatrogenic OR penis OR testicular OR testicle OR scrotal OR scrotum OR hypospadias OR congenital OR cancer OR radiation OR renal OR kidney OR dialysis OR crohn's OR diverticular OR diverticulosis OR diverticulitis OR esophageal OR behcet's OR cholelithiasis)

Limited to:

1/1/1995 to 2/8/2013

Humans

CAB Abstracts search terms: same as PubMed; search date 4/28/2013
