# Supplementary material for: Obstetric fistula in low-resource countries: an under-valued and under-studied problem – systematic review of its incidence, prevalence, and association with stillbirth
Source: BMC Pregnancy Childbirth. 2015 Aug 26;15:193. doi: 10.1186/s12884-015-0592-2 (PMC4550077; doi:10.1186/s12884-015-0592-2)
Supplement: Additional file 3: Table S1. — Obstetric fistula incidence/prevalence estimates by country [58, 21]. [file 12884_2015_592_MOESM3_ESM.docx]

## Table 1: Obstetric fistula incidence/prevalence estimates by country

| **Author (Year)** | **Country** | **Sample** | **Data Source** | **Obstetric Fistula Incidence Estimates** | **Obstetric Fistula Prevalence Estimates** | **Risk of Bias -**potential source of bias |
| --- | --- | --- | --- | --- | --- | --- |
| Jokhio (2014) [33] | Rural Sindh Province, Pakistan | 5,064 randomly selected women 15 years and older | interviews followed by gynecological exam of those reporting symptoms consistent with fistula |  | 20/5,064 **3.9** cases of OF per 1000 women > 15 years  (95% CI 2.4-6.0) 20/4,443  **4.5** cases of OF per 1000 parous women > 15 years (95% CI 2.8-6.8) | Low |
| Teghrarian (2004) [35] | Bangladesh | 31,889 ever-married women in 6 randomly selected communities | in-depth interviews, 2003 |  | **1.69** cases of OF per 1000 ever-married women  (95% CI 1.29-2.19) | Moderate - grey literature report of unpublished UNFPHA study - no physical exam |
| Bhatia (1997) [22] | Karnataka, India | 385 of 440 mothers of children 6-12 months of age from a parent study of 2,400 rural and 1,200 urban women | report of “constant leakage of feces from vagina” during social worker interview followed by gynecological exam | 2/385 by self-report 5.19 per 1000 mothers (95% CI 0.87-17.05) 1/385 by exam **2.60** per 1000 mothers  (95% CI 0.13-12.74) |  | Low |
| Fronczak (2005) [23] | Dhaka “slum” areas, Bangladesh | 557 women at 1-month post-partum of a total of 1506 post-partum women from random multistage sample | self-report of leak of urine or feces followed by  gynecological exam | 3/557 by self-report 5.39 per 1000 mothers  (95% CI 1.37-14.59) 0/557 by exam **0** per 1000 mothers (95% CI 0.0-5.37) |  | Low |
| Kulkarni (2008) [30] | Nashik District, Maharashtra, India | community-based cross-sectional study 1,167 volunteers for physical exam of 1,560 non-pregnant, ever-married, ever-pregnant 15-44-year-old women selected via stratified systematic random sample | physical exam |  | 1/1,167  **0.86** per 1000 non-pregnant, ever-married, ever-pregnant 15-44-year-old women  (95% CI 0.04-4.22) | Moderate - limited to women  < 44 years - 25% declined exam |
| Ferdous (2012) [24] | Matlab, Bangladesh | 1,162 post-partum women in HDSS** area: 624 with complicated births or perinatal death and 538 with uncomplicated births | physical exam | 0/1,162 **0** per 1000 post-partum women  (95% CI 0.0-2.6) |  | Low |
| Teghrarian (2004) [35] | India | community-based studies in 4 states 650, 385, 803, and 3,600 women | surveys, 1989-1993 |  | **4.62** cases of OF per 1000 women  (95% CI 1.18-12.51) **5.19** cases of OF per 1000 women (95% CI 0.87-17.05) **75.97** cases of OF per 1000 women  (95% CI 59.12-95.87) **3.1** cases of OF per 1000 women  (95% CI 1.61-5.30) | High - grey literature report of unpublished UNFPHA study - methodology not described |
| Tsui (2007) [4] | Nigeria | nationally representative sample of women 15-49 years old | models based on 1999 Demographic and Health Survey (DHS) prolonged labor data, UN population data, and probability of obstructed labor given prolonged labor + probability of OF given obstructed labor | projected OF incidence:  **2.11** per 1000 deliveries in women 12-49 years **4.09** per 1000 deliveries in women < 20 years |  | Moderate-High - estimated conditional probabilities |
| Prual (2000) [19] and Vangeenderhuysen (2001) [20] | West Africa: Abidjan (Côte d’Ivoire), Bamako (Mali), Niamey (Niger), Nouakchott (Mauritania), Ouagadougou (Burkina Faso), Saint-Louis (Senegal), and rural Kaolack (Senegal) | 19,342 post-partum women of 21,557 pregnant women identified in door-to-door census | Multicenter, prospective, population-based surveys and gynecologic exam | overall: **0.103** (95% CI 0-0.37) per 1,000 deliveries  urban: **0**  (95% CI 0-0.18) per 1,000 deliveries  rural: **1.24** (95% CI 0.15-4.46) per 1,000 deliveries |  | Low |
| Walraven (2001) [31] | Farafenni, The Gambia | 1056 (56.0%) of 1871 women aged 15-54 years living in one of 20 semi-randomly sampled villages in a demographic surveillance area | Survey and gynecologic exam |  | **0.95** per 1,000 women aged 15-54 years  (95% CI 0.03-5.27) 1/ 1056 | Low |
| Muleta (2007) [58] | 7 out of 11 administrative regions of Ethiopia | random multistage sampling of regions22,826 women aged 15-49 years | House-to-house survey to identify women with any problem of bowel or bladder control followed by physical exam |  | Any OF ever  **2.45** per 1,000 women 15-49 years  (95% CI 1.87-3.17) 56/22,826 Estimated number of fistula patients in rural Ethiopia is approximately 26,819 | Low |
| Biadgilign (2013) [25] | Ethiopia | 9,713 parturients of 14,070 women aged 15-49 years from two-stage stratified sampling of clusters | Probable fistula determined by asking if parous women had leakage of urine or stool from vagina following delivery |  | **10.60**/1,000  (95% CI 8.7-12.8) parturient women aged 15-49 years ever experienced uncontrollable leakage of urine or stool from vagina 103/9713 | Moderate-High - self-report of OF status - proxy measure of OF |
| Adler (2013) [32] | South Sudan (Western Bahr al-Ghazal State) | 8865 women of childbearing age estimate based on 20% of population | Key informants identified probable cases which were confirmed by physical exam |  | **0.34** per 1,000 women aged 15-49 years 3/8865 (95% CI 0.07-1.0) | Low |
| Uganda Bureau of Statistics and Macro International Inc. (2007) [26] | Uganda | multistage stratified sample | Probable fistula determined by asking if parous women had leakage of urine or stool from vagina following delivery |  | **26.4** per 1000 women aged 15-49 years reported ever experiencing uncontrollable leakage of urine or stool from vagina (95% CI 23.1-30.0) likely overestimate 225/8531 women | Moderate-High - grey literature report - self-report of OF status - proxy measure of OF |
| Mabeya (2004) [59] | West Pokot, Kenya | Recruitment of cases via community outreach | Hospital Medical Records 1999-2003 Estimated district population WRA 150,000 |  | **0.44** per 1000 WRA* (95% CI 0.34-0.56) 66 OF repairs/150,000 WRA | Moderate-High - grey literature conference proceeding - estimated reference population - relied on medical record review |
| NSO and ORC Macro (2005) [27] | Malawi | DHS  systematic sample of households within clusters | Probable fistula determined by asking if women who gave birth in past 5 years had leakage of urine or stool from vagina following most recent birth |  | **16.1** per 1000 (95% CI 13.4-19.2) 117/7272 women | Moderate-High - grey literature report  - self-report of OF status - proxy measure of OF |
| Johnson (2007) [28] | Malawi | DHS systematic sample of households within clusters | DHS interview Probable fistula determined by asking if women who gave birth in past 5 years had leakage of urine or stool from vagina following most recent birth |  | **15.6** per 1000 live births (95% CI 13.5-18.1) 183 OF/11,699 live births Lifetime prevalence of vaginal fistula symptoms in women aged 15-49 years 4.7%  (assuming all fistulas were obstetric in origin) | Moderate-High - self-report of OF status - proxy measure of OF |
| Kalilani-Phiri (2010) [29] | 9 districts in Malawi | cross-sectional population-based study with multistage random sampling, respondents reported their own or others’ OF + hospital records were reviewed  denominator estimated as 60% of female popn in 2008 Malawi census | Community survey asking if women who gave birth in past 5 years had leakage of urine or stool from vagina following a delivery or if their sisters had; also hospital record review |  | **81.0**/1000 survey respondents  (95% CI 72.1-90.8) 266/3282 respondents and  **22.9**/1000 siblings (95% CI 18.2-28.4)  75/3279 siblings reported fistula symptoms  combined estimate from all sources: lifetime prevalence  **1.6** per 1000 women, excluding repaired OF | High - excluded women with repaired OF - estimated denominator of women 12-45 years old |

*WRA = women of reproductive age

**HDSS = health and demographic surveillance site
