# Supplementary material for: Obstetric fistula in low-resource countries: an under-valued and under-studied problem – systematic review of its incidence, prevalence, and association with stillbirth
Source: BMC Pregnancy Childbirth. 2015 Aug 26;15:193. doi: 10.1186/s12884-015-0592-2 (PMC4550077; doi:10.1186/s12884-015-0592-2)
Supplement: Additional file 4: Table S2. — Obstetric fistula with stillbirth data [59–86]. [file 12884_2015_592_MOESM4_ESM.docx]

**Table 2:**

Obstetric fistula with stillbirth data

| **Article** | **Country** | **Fistula cases, obstetric etiology** | **% of fistula cases with stillbirth or perinatal death** | **Comments** |
| --- | --- | --- | --- | --- |
| Kumar (2009) [39] | India | 317 | 90% | 80% labored at home; 65% attended by skilled personnel |
| Singh (2009) [38] | India | 389 | 39% | Includes 29 deaths in the first month. Unclear if denominator is all participants with OF, or only those who delivered vaginally. |
| Barone (2012) [36] | Bangladesh Guinea Nigeria Niger Uganda | 1,243 | 88% | Includes 46 early neonatal deaths. |
| Nathan (2009) [60] | Benin | 37 | 95% |  |
| Tebeu (2009) [61] | Cameroon | 42 | 83% |  |
| Tebeu (2012) [62] | Cameroon | 38 | 74% |  |
| Benfield (2011) [63] | Democratic Republic of the Congo | 57 | 88% |  |
| Onsrud (2011) [46] | DR Congo | 440 | 92% | Stillbirth occurred in 95% of vaginal and 87% of cesarean deliveries |
| Turan (2007) [64] | Eritrea | 26 | 100% |  |
| Browning (2007) [65] | Ethiopia | 51 | 98% |  |
| Goh (1998) [66] | Ethiopia | 110 | 95.5% |  |
| Browning (2006) [67] | Ethiopia | 481 | 95.2% |  |
| Browning (2010) [68] | Ethiopia | 489 | 94% |  |
| Kelly (1995) [69] | Ethiopia | 300 | 93% |  |
| Muleta (2010) [34] | Ethiopia | 14,822 | 92% | Cases accrued between 1974-2008. 434 cases reported baby’s sex; 91.1% of males and 78.9% of females were stillborn. |
| Gessessew (2003) [70] | Ethiopia | 184 | 88.6% |  |
| Khisa (2012) [71] | Kenya | 8 | 88% |  |
| McFadden (2011) [72] | Kenya | 77 | 87% | Includes 11 early neonatal deaths. |
| Mabeya (2004) [59] | Kenya | 64 | 73% | Grey literature. Includes 3 early neonatal deaths. |
| Weston (2011) [47] | Kenya | 70 | 64% |  |
| Hawkins (2013) [43] | Kenya | 303 | 76% | Includes 8 neonatal deaths; excludes 180 births with unknown outcome. |
| Roka (2013) [73] | Kenya | 70 | 78.6% |  |
| Raassen (2008) [74] | Kenya, Tanzania and Uganda | 579 | 88% | Includes 18 neonatal deaths in first week. Stillbirth occurred in 90% of vaginal and 87% of cesarean deliveries. |
| Rijken (2007) [75] | Malawi | 379 | 87% | Includes 23 early neonatal deaths. . |
| Savan (2010) [41] | Niger | 21 | 92% of 24 | 24 fistula cases total, 21 of obstetric etiology, 3 traumatic. Stillbirth numbers reported as outcome of "most recent delivery" not specified if associated with birth causing fistula and included traumatic cases. |
| Alio (2011) [76] | Niger | 20 | 100% | Stillborn or neonatal death within 2 days of birth |
| Nafiou (2007) [77] | Niger | 111 | 100% | Perinatal deaths |
| Meyer (2007) [78] | Niger | 58 | 97% |  |
| Cam (2010) [79] | Niger | 51 | 61% |  |
| Roenneburg (2006) [44] | Niger | 56 | 89% |  |
| Wall (2004) [37] | Nigeria | 899 | 92% | In addition, of 75 live births, 14 died within the first 4 weeks, most within 7 days. |
| Orji (2007) [80] | Nigeria | 68 | 90% |  |
| Hilton (1998) [81] | Nigeria | 2202 | 90% | Stillbirth occurred in 97% of spontaneous vaginal, 89% of assisted vaginal, and 77% of cesarean deliveries |
| Ezegwui (2005) [82] | Nigeria | 68 | 84% | Includes 4 early neonatal deaths |
| Kirby (2012) [42] | Nigeria | 83 | 66% | 55 of the 83 participants had a history of a stillborn child, but it was not specified if it was experienced with the same birth that caused the fistula. |
| Tunçalp (2014) [50] | Nigeria | 50 and 29 | 84% and 74% | 80% and 56% of OF cases were delivered by Caesarean section |
| Kamara (2012) [83] | Sierra Leone/Aberdeen Women's Centre | 641 | 95.2% | Unpublished dissertation. |
| Ramphal (2007) [84] | South Africa | 41 | 93% |  |
| Ramphal (2008) [85] | South Africa | 41 | 87% |  |
| Mohamed (2009) [86] | Sudan | 47 | 89% |  |
| Pope (2011) [53] | Tanzania | 25 | 88% |  |
| Kayondo (2011) [87] | Uganda | 77 | 90% |  |
| Bangser (2011) [88] | Uganda and Tanzania | 124 | 90% |  |
| Holme (2007) [40] | Zambia | 237 | 78% | Stillbirth numbers were reported as outcome of most recent delivery not reported if it was the same birth associated with fistula. |

* Addressed in comments
